# Supplementary material for: Single-Cell Heterogeneity of Epigenetic Factor Regulation Deciphers Alteration of RNA Metabolism During Proliferative SHH-Medulloblastoma
Source: Cancers (Basel). 2025 Oct 24;17(21):3424. doi: 10.3390/cancers17213424 (PMC12609976; doi:10.3390/cancers17213424)
Supplement: Supplementary file 1 [file cancers-17-03424-s001.zip › cancers-3890554-supplementary.pdf]

Supplementary Materials

Single-Cell Heterogeneity of Epigenetic Factor Regulation Deciphers Alteration of RNA Metabolism During Proliferative SHH-Medulloblastoma

Supplemental Figures

Supplemental Figure S1: Preprocessing of medulloblastoma tumor RNA-sequencing from PBTA cohort; Supplemental Figure S2: Regulation of preditive epifactors in PBTA-medulloblastoma according molecular subtype classification; Supplemental Figure S3: Preprocessing of medulloblastoma tumor RNA-sequencing from Willamson cohort; Supplemental Figure S4: Regulation of preditive epifactors in Williamson-medulloblastoma according molecular subtype classification; Supplemental Figure S5: Epifactors overall survival analyses in PBTA-MB RNA-sequencing cohort

Supplemental Tables

Supplemental Table S1: Machine learning predictive scores for molecular subtypes obtained with the 62 Epifactors on PBTA medulloblastoma cohort

Supplemental Table S2: General additive model p-values for best seventy markers identified on proliferative SHH-MB malignant cells

Supplemental Figures

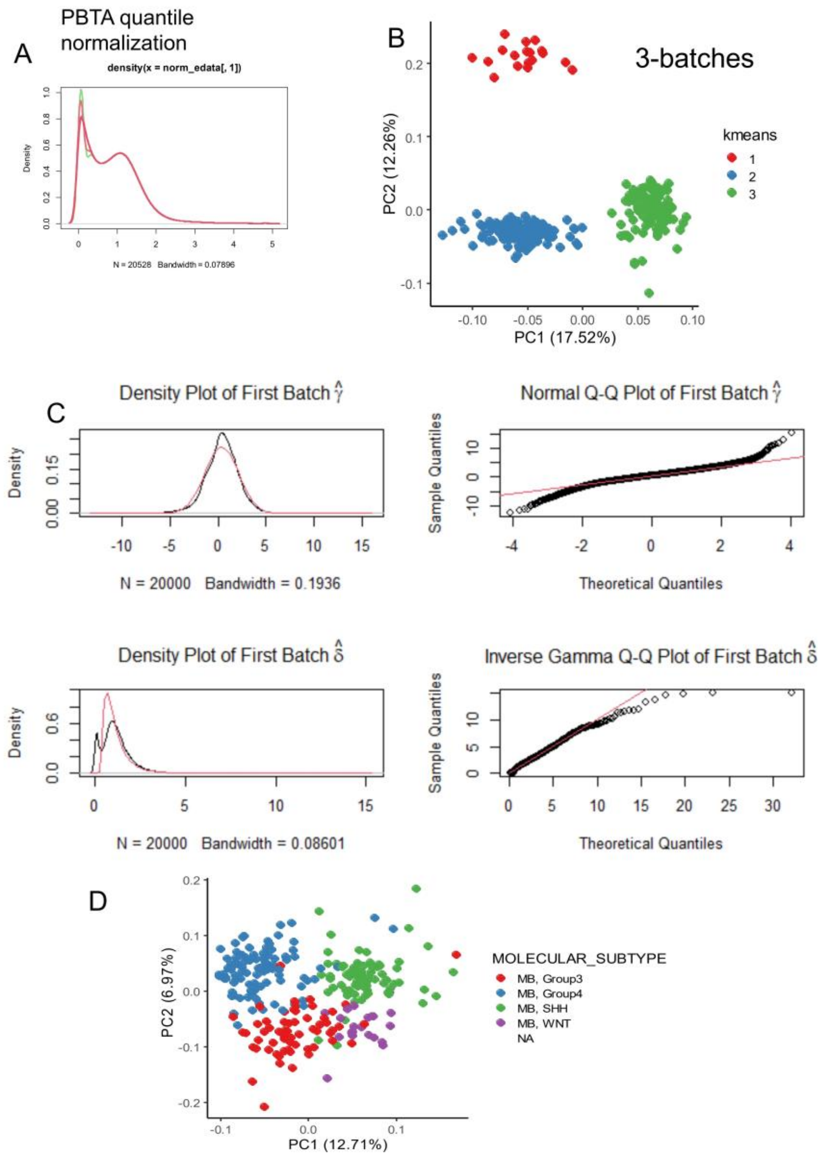

Supplementary Figure S1. Preprocessing of medulloblastoma tumor RNA-sequencing from the PBTA cohort. **A**. Density plot of quantile-normalized RNA-sequencing counts after positive mean-expression filtering (n = 20 528 genes). **B**. Principal component analysis (PCA) of the whole transcriptome showing identification of three technical batches by *k-means* clustering. **C**. Batch correction of PBTA medulloblastoma transcriptomes using the *ComBat* algorithm. **D**. Unsupervised PCA of *ComBat*-corrected data with molecular-subtype stratification demonstrating effective batch removal.

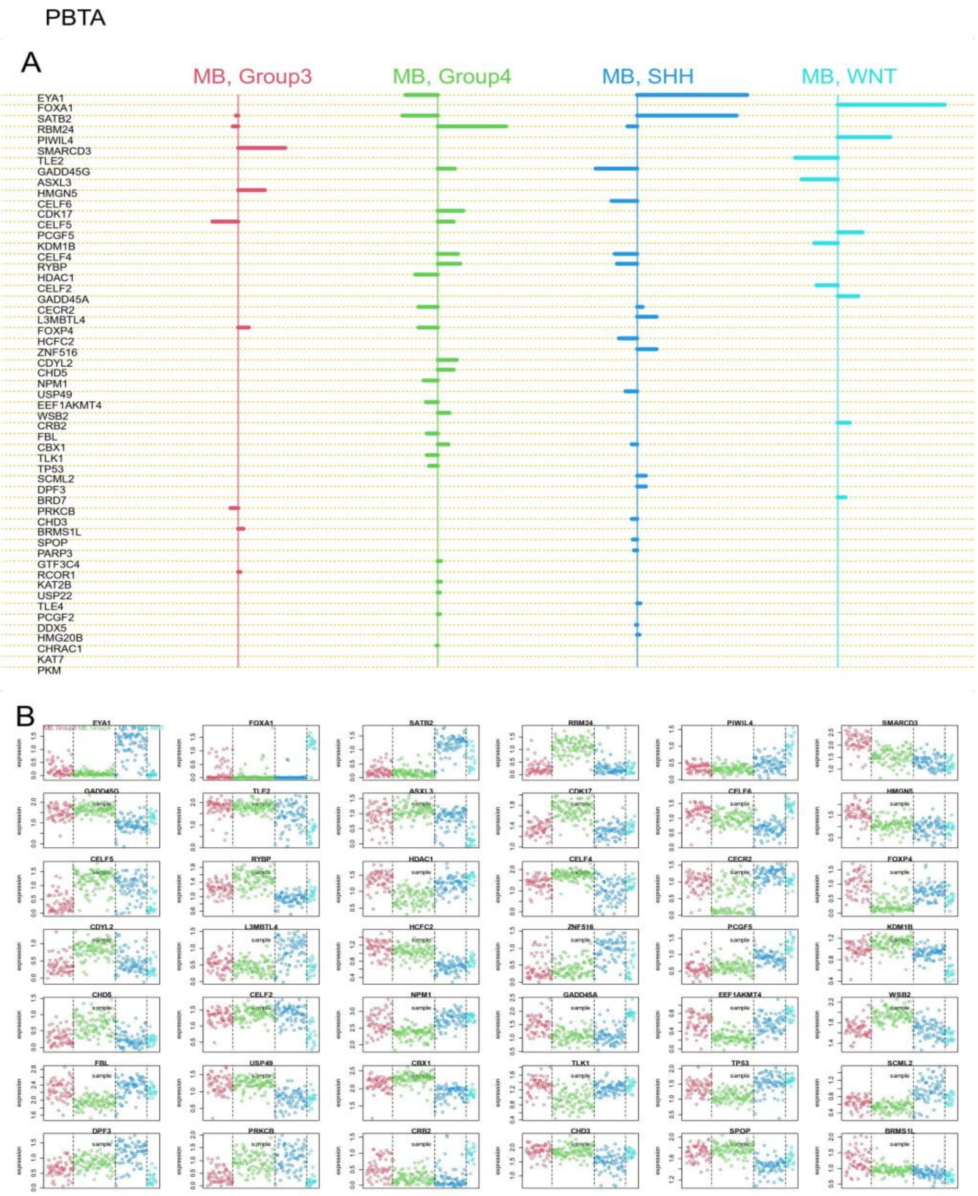

Supplementary Figure S2. Regulation of predictive epifactors in PBTA medulloblastoma according to molecular subtype **A**. Bar plot of machine-learning predictive scores (from the PAMR classifier) for epifactors across molecular subtypes in the PBTA cohort. **B**. Expression plots showing individual predictive epifactor expression levels stratified by molecular subtype.

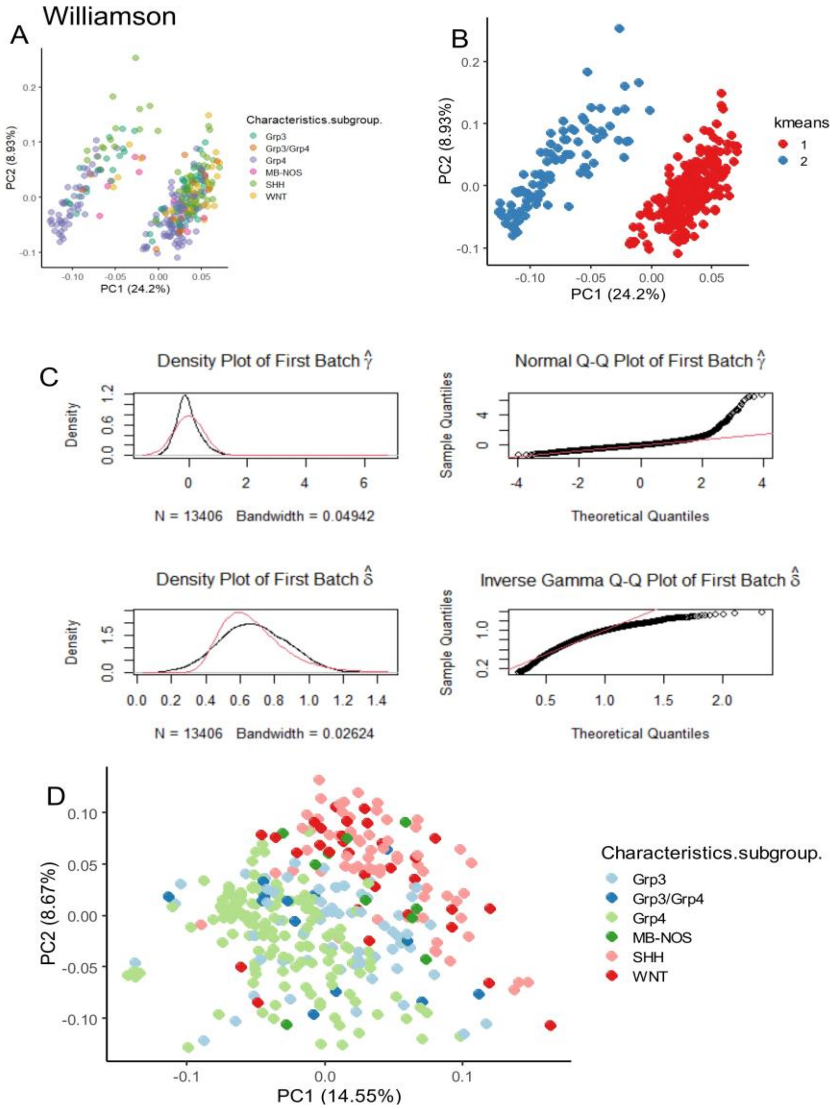

Supplementary Figure S3. Preprocessing of medulloblastoma tumor RNA-sequencing from the Williamson cohort **A**. PCA of the whole transcriptome before batch normalization, colored by molecular subtype. **B**. PCA identifying two technical batches by *k-means* clustering. **C**. Batch correction using the *ComBat* algorithm. **D**. Unsupervised PCA after correction showing effective batch removal and correct molecular-subtype segregation.

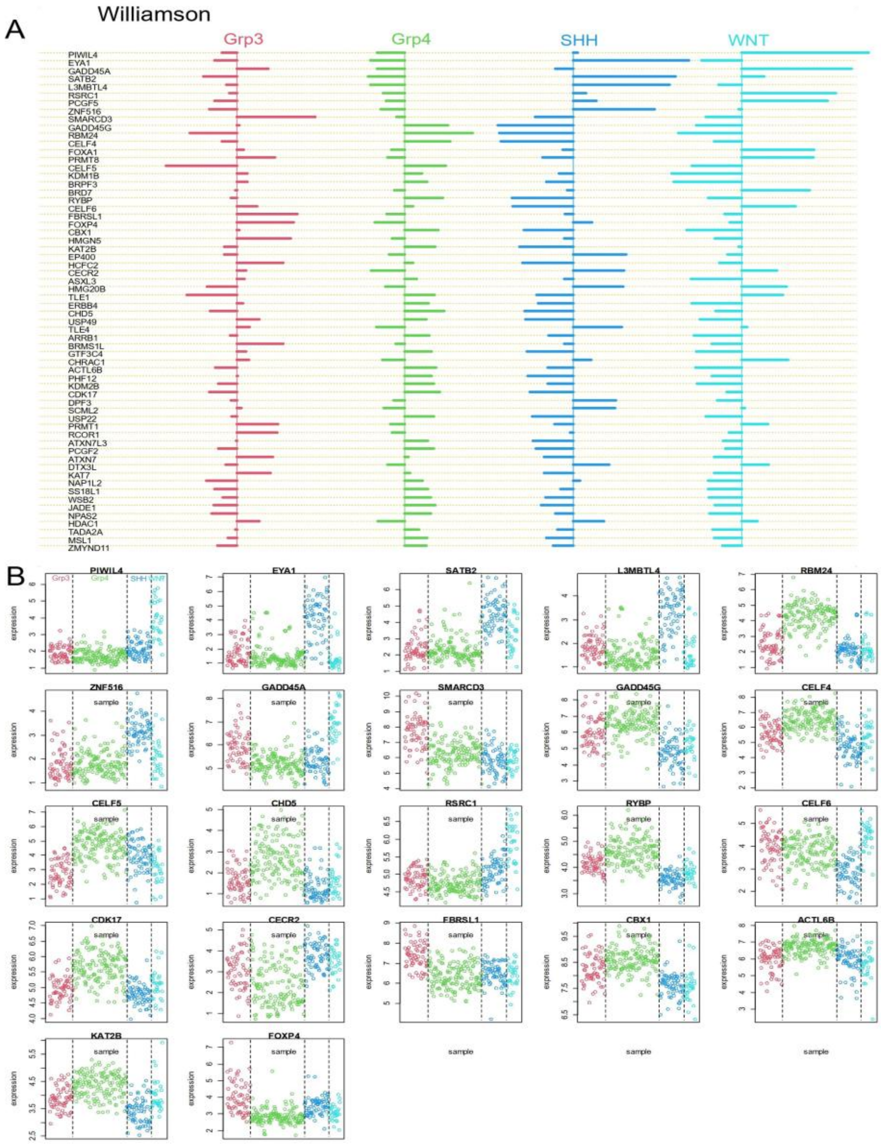

Supplementary Figure S4. Regulation of predictive epifactors in Williamson medulloblastoma according to molecular subtype **A**. Bar plot of PAMR-based predictive scores for epifactors across molecular subtypes in the Williamson cohort. **B**. Expression plots displaying individual predictive epifactor levels per molecular subtype.

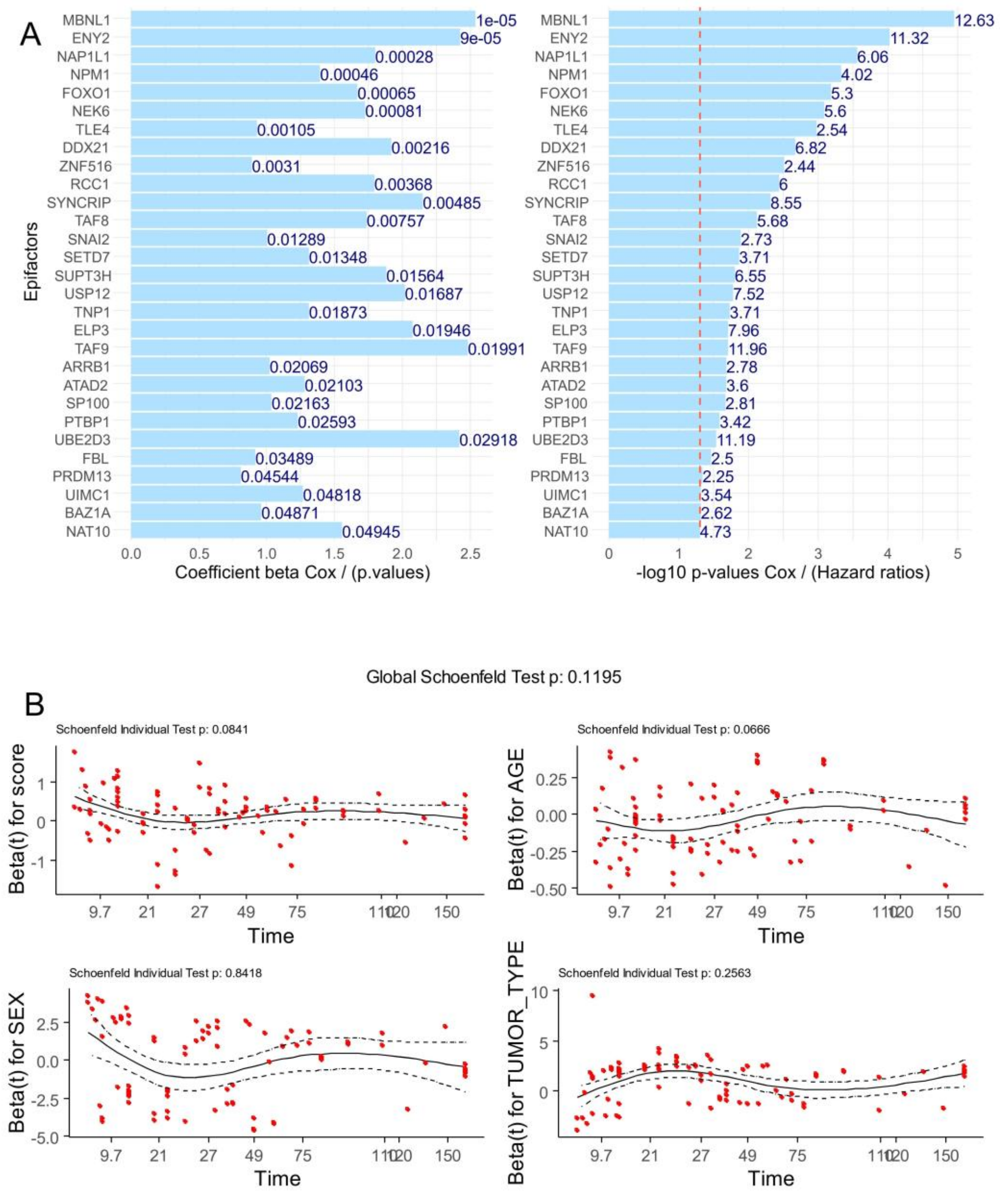

Supplementary Figure S5. Epifactor overall-survival analyses in PBTA medulloblastoma RNA-sequencing cohort **A**. Bar plot of univariate Cox proportional-hazards analyses for the twenty adverse epifactors, showing hazard ratios and significance for overall survival. **B**. Global and individual Schoenfeld residual tests for each covariate in the multivariable Cox model, verifying the proportional-hazards assumption.

Supplemental Tables

Supplemental Table S1: Machine learning predictive scores for molecular subtypes obtained with the 62 Epifactors on PBTA medulloblastoma cohort (value : pamr predictive score)

Value	Gene	Rank	Group
0.5864	SMARCD3	7	Group3
0.383	HMGN5	14	Group3
0.0224	HDAC1	21	Group3
0.0038	HCFC2	23	Group3
0.2244	FOXP4	27	Group3
0.1699	BRMS1L	41	Group3
0.1425	RCOR1	46	Group3
0.0914	KAT7	57	Group3
0.0851	FBRSL1	62	Group3
0.0597	ATXN7	70	Group3
0.0517	PRMT1	76	Group3
0.7627	RBM24	4	Group4
0.0198	ASXL3	8	Group4
0.2481	GADD45G	9	Group4
0.0116	KDM1B	11	Group4
0.2343	CELF5	16	Group4
0.3363	CDK17	17	Group4
0.2859	CELF4	18	Group4
0.3071	RYBP	20	Group4
0.2651	CDYL2	28	Group4
0.2408	CHD5	29	Group4
0.0462	USP49	31	Group4
0.0025	BRPF3	32	Group4
0.1949	WSB2	34	Group4
0.1857	CBX1	36	Group4
0.029	ARRB1	47	Group4
0.1121	GTF3C4	55	Group4
0.108	KAT2B	56	Group4
0.1038	USP22	58	Group4
0.0986	PCGF2	60	Group4
0.0592	ERBB4	67	Group4
0.0579	KDM2B	71	Group4
0.0576	JADE1	72	Group4
0.0539	ACTL6B	74	Group4
0.0526	NPAS2	75	Group4
0.0415	ATXN7L3	77	Group4
0.0001	PHF12	79	Group4
0.0349	SS18L1	83	Group4
0.0271	TLE1	85	Group4
0.0255	NAP1L2	86	Group4
0.0112	TADA2A	92	Group4
0.0087	ZMYND11	93	Group4
0.0065	MSL1	94	Group4
1.1968	EYA1	2	SHH
1.0902	SATB2	3	SHH

0.2882	L3MBTL4	22	SHH
0.288	ZNF516	24	SHH
0.1434	CECR2	26	SHH
0.1832	SCML2	39	SHH
0.1796	DPF3	40	SHH
0.1226	TLE4	50	SHH
0.115	HMG20B	53	SHH
0.0899	DTX3L	61	SHH
0.0361	EP400	82	SHH
1.283	FOXA1	1	WNT
0.7387	PIWIL4	5	WNT
0.4506	PCGF5	10	WNT
0.4163	GADD45A	13	WNT
0.0015	CELF6	15	WNT
0.3237	CRB2	19	WNT
0.2809	BRD7	25	WNT
0.1506	RSRC1	44	WNT
0.1298	CHRAC1	49	WNT
0.1136	PRMT8	54	WNT

Supplemental Table S2: General additive model *p*-values for best seventy markers identified on proliferative SHH-MB malignant cells

gam_pvalues	Rank	gene_symbol
4.06E-154	1	TOP2A
1.58E-149	2	HMGB2
1.24E-144	3	CENPF
4.26E-137	4	MKI67
1.58E-128	5	SMC4
1.14E-126	6	NUSAP1
1.57E-124	7	ASPM
4.86E-82	8	TUBA1B
1.63E-81	9	HMGN2
1.92E-81	10	HMGB1
1.12E-79	11	DEK
6.93E-78	12	UBE2C
3.11E-77	13	TYMS
3.71E-75	14	H2AFZ
8.73E-73	15	TMPO
1.22E-72	16	NUCKS1
4.81E-71	17	NASP
3.27E-70	18	PCNA
9.85E-70	19	HNRNPA2B1
1.88E-64	20	PRKDC
1.56E-63	21	FUS
1.02E-62	22	RAD21
2.07E-62	23	PCLAF
4.07E-62	24	HIST1H4C
1.83E-59	25	DUT
3.96E-57	26	CBX5
4.26E-56	27	CBX3
2.93E-55	28	HNRNPD
5.62E-55	29	TUBB4B
3.36E-54	30	SUPT16H
1.91E-53	31	PSIP1
9.15E-53	32	MCM7
1.30E-52	33	HIST1H1D
1.68E-52	34	HELLS
7.34E-51	35	SRSF2
9.20E-51	36	H2AFV
3.00E-50	37	PAICS
7.24E-50	38	ANP32E
8.94E-50	39	ARL6IP1
3.18E-49	40	RAN
5.65E-49	41	SOX2
1.23E-48	42	SRSF7
1.90E-47	43	SMC1A
2.51E-47	44	SYNE2
6.27E-47	45	KPNB1
6.64E-47	46	B2M
2.09E-46	47	HNRNPA3

4.89E-46	48	RANBP1
1.22E-45	49	SNRPB
1.66E-45	50	LMO4
1.79E-45	51	SRSF10
1.96E-45	52	NAP1L1
3.34E-45	53	HSPD1
5.96E-45	54	PARP1
2.07E-44	55	UQCC2
1.32E-43	56	BOC
1.35E-43	57	HNRNPU
1.59E-43	58	NCL
1.68E-43	59	PPIA
7.97E-43	60	SERBP1
1.51E-42	61	SRSF3
2.94E-42	62	HHIP
3.36E-42	63	CNTLN
4.48E-42	64	HP1BP3
6.04E-42	65	SNRPG
1.37E-41	66	HNRNPAB
1.64E-41	67	RBMX
2.26E-41	68	MSH6
2.73E-41	69	HNRNPM
3.29E-41	70	RIF1
